# Special Issue “Bioinformatics Study in Human Diseases: Integration of Omics Data for Personalized Medicine”

**DOI:** 10.3390/ijms251910579

**Published:** 2024-10-01

**Authors:** Hung-Yu Lin, Pei-Yi Chu

**Affiliations:** 1 Department of Post-Baccalaureate Medicine, College of Medicine, National Chung Hsing University, Taichung 402, Taiwan; 2 Research Assistant Center, Show Chwan Memorial Hospital, Changhua 500, Taiwan; 3 Department of Pathology, Show Chwan Memorial Hospital, Changhua 500, Taiwan; 4 National Institute of Cancer Research, National Health Research Institutes, Tainan 704, Taiwan

The field of bioinformatics has made remarkable strides in recent years, revolutionizing our approach to understanding and treating human diseases. This Special Issue, “Bioinformatics Study in Human Diseases: Integration of Omics Data for Personalized Medicine”, showcases cutting-edge research that harnesses the power of bioinformatics and multi-omics integration to advance precision medicine.

The convergence of multiple scientific disciplines and technological advances has led to unprecedented growth in the field of multi-omics. This surge is evidenced by the more than doubling of multi-omics scientific publications within just two years (2022–2023) since its first referenced mention in 2002, as indexed by the National Library of Medicine [1]. Multi-omics approaches have demonstrated their capability to provide comprehensive insights into complex biological systems, representing a transformative force in health diagnostics and therapeutic strategies [2].

The 12 papers published in this issue demonstrate the breadth and depth of bioinformatics applications in various areas of medical research. From metabolic modeling to transcriptomics, these studies exemplify how computational approaches can unravel complex biological mechanisms and identify potential biomarkers and therapeutic targets.

The COVID-19 pandemic has highlighted the need for rapid and comprehensive bioinformatic analyses in understanding disease mechanisms. A study on COVID-19-induced pulmonary fibrosis demonstrates how bioinformatics can shed light on complex pathological processes. By analyzing publicly available data, the researchers show that COVID-19 infection drives endothelial lineage cells towards myofibroblasts, contributing to pulmonary fibrosis. This finding provides crucial insights into the long-term effects of COVID-19 and potential therapeutic targets [3].

One of the highlights is the innovative pipeline for integrating genome-scale metabolic models with patient plasma metabolome data, offering new insights into endothelial metabolism in trauma patients [4]. This approach aligns with the growing trend of integrating multiple omics layers to gain a more comprehensive understanding of biological systems [5,6].

In the realm of cancer research, several studies have made significant contributions. The computational analysis of FLT3 variations in acute myeloid leukemia provides valuable insights into the structural and functional impacts of mutations, potentially guiding future experimental and clinical studies [7]. Another study on cervical cancer elucidates the role of LAMB3 in promoting cancer cell migration, invasion, and survival [8]. These studies exemplify how multi-omics approaches can facilitate early disease detection and prevention, in addition to aiding in the discovery of crucial biomarkers for diagnosis, prognosis, and treatment monitoring [9,10].

Network-based approaches are applied in periodontitis research to identify master regulator transcription factors [11]. This aligns with the broader trend of using sophisticated computational utilities and stringent statistical methodologies to ensure accurate data interpretation in multi-omics research [12].

Several studies focus on the microbiome and its interactions with human health [13,14]. These studies contribute to the growing body of research recognizing the importance of the microbiome in multi-omics analyses and its impact on human health [15,16].

The exploration of epigenetic mechanisms in Alzheimer’s disease offers new perspectives on neurodegenerative disorders [17]. This research aligns with the broader goal of using multi-omics to provide a deep understanding of disease-associated molecular mechanisms [18,19].

In reproductive health, single-cell analysis reveals insights into testicular inflammation in idiopathic non-obstructive azoospermia [20]. This exemplifies the power of integrating various omics technologies to gain comprehensive insights into complex biological systems [21,22].

The application of supervised learning and multi-omics integration in kidney renal clear cell carcinoma research demonstrates the clinical significance of inner membrane mitochondrial protein (IMMT) in prognostic prediction and understanding the tumor immune microenvironment [23]. This study showcases how multi-omics can not only facilitate precision medicine by accounting for individual omics profiles [24,25] but also bridge the gaps between mitochondrial medicine and clinical oncology [26].

Other notable contributions include the identification of potential biomarkers for methamphetamine use disorder [27] and the elucidation of TGF-β signaling in saphenous vein graft failure [28], both of which have significant implications for personalized medicine and are in line with specific clinical scopes [29,30].

While multi-omics offers substantial promise, its research and large-scale application in healthcare present several challenges. These include cohesively integrating and normalizing data across varied omics platforms, managing the sheer volume and high dimensionality of multi-omics datasets, and addressing the ethical implications of managing sensitive health information [31,32].

For multi-omics to truly revolutionize healthcare, it demands rigorous validation, tangible real-world applications, and smooth integration into existing healthcare infrastructures. Key developments include targeted sampling methods, the use of artificial intelligence in formulating health indices, the integration of sophisticated n-of-1 statistical models such as digital twins, and the incorporation of blockchain technology for heightened data security [33,34].

As we move forward, the continued development and application of these computational approaches will undoubtedly play a crucial role in shaping the future of precision medicine. The field is poised to overcome the current challenges and realize its full potential in revolutionizing personalized healthcare [35,36].
